# Non-invasive detection of iron deficiency by fluorescence measurement of erythrocyte zinc protoporphyrin in the lip

**DOI:** 10.1038/ncomms10776

**Published:** 2016-02-17

**Authors:** Georg Hennig, Christian Homann, Ilknur Teksan, Uwe Hasbargen, Stephan Hasmüller, Lesca M. Holdt, Nadia Khaled, Ronald Sroka, Thomas Stauch, Herbert Stepp, Michael Vogeser, Gary M. Brittenham

**Affiliations:** 1 Laser-Forschungslabor, LIFE-Zentrum, Klinikum der Universität München, Feodor-Lynen-Strasse 19, 81377 Munich, Germany; 2 Perinatalzentrum Großhadern, Klinikum der Universität München, Marchioninistrasse 15, 81377 Munich, Germany; 3 Institut für Laboratoriumsmedizin, Klinikum der Universität München, Marchioninistrasse 15, 81377 Munich, Germany; 4 Nestlé Research Center, Nestec Ltd., PO Box 44, 1000 Lausanne 26, Switzerland; 5 Deutsches Kompetenz-Zentrum für Porphyriediagnostik und Konsultation, MVZ Labor PD Dr. Volkmann und Kollegen GbR, Kriegsstrasse 99, 76133 Karlsruhe, Germany; 6 Department of Pediatrics, Columbia University, Children's Hospital of New York, Room CHN 10-08, 3959 Broadway, New York, New York 10032, USA

## Abstract

Worldwide, more individuals have iron deficiency than any other health problem. Most of those affected are unaware of their lack of iron, in part because detection of iron deficiency has required a blood sample. Here we report a non-invasive method to optically measure an established indicator of iron status, red blood cell zinc protoporphyrin, in the microcirculation of the lower lip. An optical fibre probe is used to illuminate the lip and acquire fluorescence emission spectra in ∼1 min. Dual-wavelength excitation with spectral fitting is used to distinguish the faint zinc protoporphyrin fluorescence from the much greater tissue background fluorescence, providing immediate results. In 56 women, 35 of whom were iron-deficient, the sensitivity and specificity of optical non-invasive detection of iron deficiency were 97% and 90%, respectively. This fluorescence method potentially provides a rapid, easy to use means for point-of-care screening for iron deficiency in resource-limited settings lacking laboratory infrastructure.

## Abstract (web summary)

Iron deficiency, the most common health problem in the world, has required a blood test for diagnosis. Here, the authors show that iron deficiency can be detected non-invasively and quickly by measuring the fluorescence of red blood cell zinc protoporphyrin in the microcirculation of the lip.

## Graphical abstract

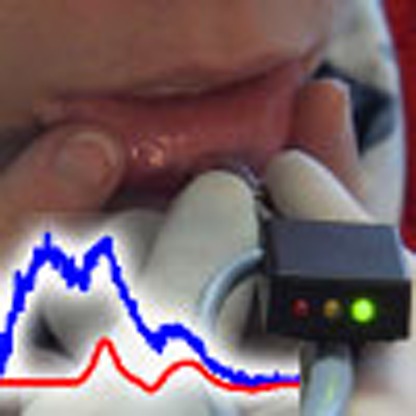

Worldwide, iron deficiency affects more individuals than any other health problem^1^. Lack of iron causes anaemia^2^, impairs cognitive and behavioural development in childhood^3^,^4^, compromises immune responsiveness^5^, diminishes physical performance^6^ and, when severe, increases mortality among infants, children and pregnant women^4^,^7^. Effective and inexpensive treatment is available^3^, but most of those affected are unaware of their need for more iron. Detection of iron deficiency, with a requirement for a blood sample and some forms of laboratory processing^8^, has been generally unavailable in the same resource-limited settings where a lack of iron is most common^9^.

We report an optical method to detect iron deficiency without the need for a blood sample. In the developing red blood cell, the insertion of iron into protoporphyrin IX is the final step in the production of haem for incorporation into haemoglobin^10^. If iron is unavailable, divalent zinc is incorporated instead, producing zinc protoporphyrin, which persists for the life of the red blood cell as a biochemical indicator of functional iron deficiency^11^,^12^. In regions endemic for malaria and other infections, the World Health Organization recommends measurement of the red blood cell zinc protoporphyrin as the preferred indicator to screen children for iron deficiency^13^. In the United States, the American Academy of Pediatrics recommends universal screening for iron deficiency at 1 year of age^14^, and the use of red blood cell zinc protoporphyrin for this purpose has been suggested^15^. Screening for iron deficiency using red blood cell zinc protoporphyrin meets the classical criteria defined by Wilson and Jungner^16^ as well as more recently proposed standards^17^. Non-invasive screening is likely to be more acceptable to children and many other populations than methods requiring finger- or venipuncture.

With blue light excitation, zinc protoporphyrin fluoresces, while haem does not. To detect this faint fluorescence, we use an optical fibre probe to illuminate and acquire the fluorescence emission spectra from the lower lip, where only a thin, non-pigmented epithelial layer covers the blood-filled capillaries perfusing the underlying tissue. Still, this mucosal tissue contains collagen, elastin and other fluorophores that produce a background fluorescence whose intensity is two orders of magnitude greater than that from red blood cell zinc protoporphyrin. We use dual-wavelength excitation and spectral fitting to distinguish the faint zinc protoporphyrin fluorescence from the mucosal tissue background. Spectral analysis is also used to identify tissue sites suitable for quantitative fluorescence measurements. Here we describe the integration of these technical innovations in portable, point-of-care instrumentation to quantify red blood cell zinc protoporphyrin fluorescence from the lower lip. Measurements use only light and subjects have no electrical exposure. Using the portable instrument, we sought to compare the results of fluorescence measurements *in vivo* in women shortly after childbirth with determinations on blood samples *in vitro* by a standard reference method using high-performance liquid chromatography (HPLC), and by a conventional haematofluorometer that measures red blood cell zinc protoporphyrin fluorescence directly from a blood sample. Optical measurements in the lower lip in these women have a sensitivity of 97% and a specificity of 90% for iron deficiency. Overall, our results provide proof-of-concept validation for non-invasive detection of iron deficiency by optical fibre probe fluorescence spectroscopy of the lower lip.

## Results

**Figure 1:**
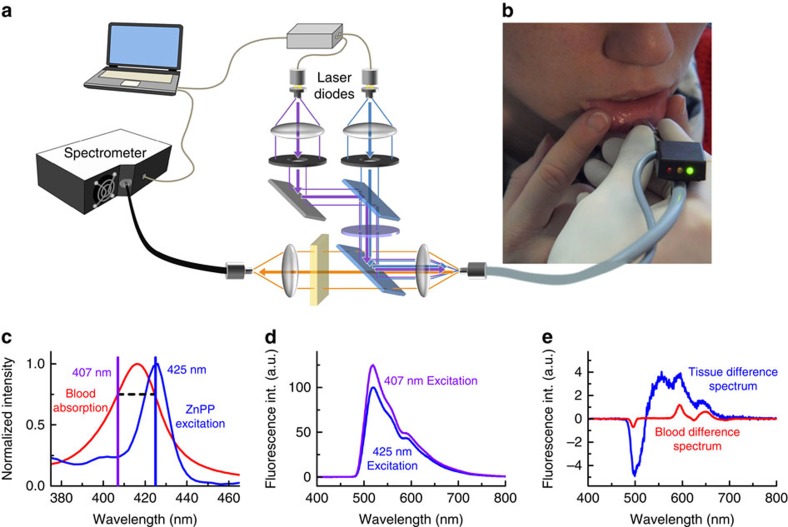
Tissue fluorescence measurements using dual-wavelength excitation. (**a**) Simplified schematic of the set-up. Light of two laser diodes at 407 and 425 nm is superimposed and coupled into an optical fibre for fluorescence excitation. The generated fluorescence is collected by the same fibre and detected with a spectrometer. (**b**) Photograph of the measurement procedure with the fibre-optic probe placed on the lower lip. An adequate blood absorption index is indicated by the green light on the black indicator box. (**c**) The fluorescence excitation maximum of red blood cell zinc protoporphyrin (ZnPP) is at 425 nm. The second excitation wavelength of 407 nm is chosen for identical blood absorption. (**d**) Typical fluorescence spectra of the oral mucosa of a single subject with elevated ZnPP/haem ratio when excited with 425 and 407 nm. (**e**) Difference spectrum of the spectra in **d** with normalized 407-nm spectrum, resulting in background reduction of ∼93%. The corresponding difference spectrum of a measurement in whole blood using the same instrumentation *in vitro* is shown in red, obtained by placing the optical fibre in a sample of whole blood.

In brief, to measure non-invasively the red blood cell zinc protoporphyrin, we place the optical fibre probe in gentle contact with the red vermillion of the lower lip of the subject and slowly move the probe over the surface (Fig. 1a,b). Suitable sites for measurement of the red blood cell zinc protoporphyrin are detected in real time by determination of the ‘blood absorption index' and indicated to the examiner by illumination of an light-emitting diode (LED) indicator attached to the optical fibre probe (Fig. 1b). The blood absorption index, detailed below, identifies sites on the lower lip where mucosal tissue properties (including haemoglobin concentration, light scattering coefficient, blood vessel size and epithelial thickness) permit quantitative measurement of red blood cell zinc protoporphyrin. While the blood absorption index identifies suitable sites for measurement, the index does not enter into the calculation. Instead, dual-wavelength excitation and spectral fitting are then used at a suitable site to quantitatively determine zinc protoporphyrin. In presenting our results below, we first describe the dual-wavelength and spectral fitting procedures and then detail how the blood absorption index can identify suitable tissue sites for quantitative measurements.

### Measurement of erythrocyte zinc protoporphyrin fluorescence

**Figure 2:**
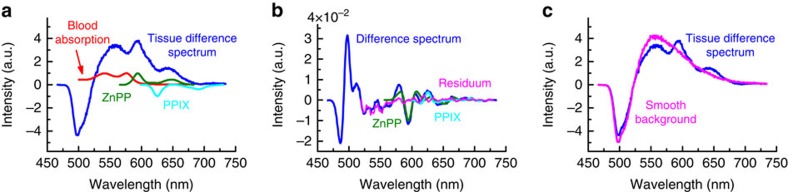
Spectral fitting procedure to separate the ZnPP and protoporphyrin IX (PPIX) fluorescence from background. (**a**) Averaged difference spectrum of 100 single spectra measured in the same subject as for Fig. 1e, and the input spectra of ZnPP, PPIX and blood absorption that are used for the fitting procedure. (**b**) Second derivatives of the tissue difference spectrum shown in **a**, and of the fitted components. (**c**) Difference spectrum and the smooth background that remains when ZnPP, PPIX and blood absorption are removed according to the obtained fit amplitudes, indicating the effectiveness and validity of the spectral fitting procedure.

To detect the faint fluorescence of red blood cell zinc protoporphyrin, we use an optical fibre probe to illuminate and acquire the fluorescence emission spectra from the blood-filled capillaries beneath the wet vermillion of the lower lip (Fig. 1a,b; see Supplementary Figs 1 and 2 for a detailed schematic and a photograph of the instrument with the optical fibre probe). At the wavelength of maximum red blood cell zinc protoporphyrin fluorescence excitation, 425 nm, collagen, elastin and other tissue fluorophores emit background fluorescence with an intensity a hundredfold greater than that from red blood cell zinc protoporphyrin^18^. To quantitatively extract the zinc protoporphyrin fluorescence in the presence of this very large background, we acquire additional fluorescence spectra excited at a wavelength of 407 nm (ref. 19). Both spectra are obtained with an excitation power of ∼2.5 mW for the illuminated area of 1-mm diameter to comply with the light-exposure limits defined by the international standard IEC 60825-1:2014. At the 425- and 407-nm excitation wavelengths, the light absorption of capillary blood is virtually identical and, under the conditions of our measurements, little influenced by tissue properties (Fig. 1c; see Supplementary Note 1 for details of the choice of the excitation wavelengths and the influence of tissue properties). Because structures in the tissue absorption spectrum are dominated by capillary blood in this wavelength region and light scattering is nearly identical^20^, both wavelengths probe approximately the same tissue volume, producing similar background fluorescence spectra (Fig. 1d). Since the excitation efficiency of red blood cell zinc protoporphyrin is ∼78% lower at the 407-nm excitation wavelength compared with that at 425 nm (Fig. 1c), the difference spectrum retains most of the zinc protoporphyrin fluorescence while reducing the fluorescence background at 593 nm by 93±2% (s.d.; Fig. 1e). At 407 nm, protoporphyrin IX fluorescence excitation is greater than that at 425 nm, producing a negative contribution to the difference spectrum. To extract the zinc protoporphyrin fluorescence contribution from the difference spectrum, a spectral fitting algorithm is applied (Fig. 2). Because the spectral shape of the fluorescence background varies from subject to subject and in different areas on the lower lip within the same subject, fully modelling the difference spectrum proved impractical. We use an alternative approach based on calculating the second derivative of the difference spectrum^21^. This procedure is insensitive to the background fluorescence spectrum, which lacks characteristic structures. Inputs for the fitting algorithm are the blood absorption spectrum and the zinc protoporphyrin and protoporphyrin IX fluorescence spectra (see Supplementary Figs 3 and 4 for detailed spectra). The output parameters are the amplitudes of the zinc protoporphyrin and protoporphyrin IX fluorescence spectra and of the blood absorption spectrum.

### Identification of suitable measurement sites

**Figure 3:**
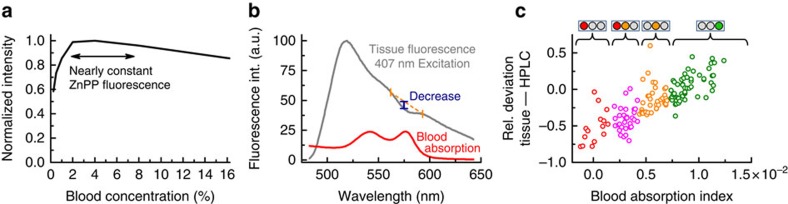
Using the blood absorption index to identify suitable tissue sites for quantitative measurement of the ZnPP/haem ratio. (**a**) For whole-blood concentrations between 2 and 8% (whole blood of a healthy subject diluted by saline), the measured ZnPP fluorescence remains little changed despite this large variation in the blood concentration. (**b**) Illustration of the blood absorption index. The magnitude of the decrease in the tissue-background fluorescence spectrum resulting from blood absorption, termed the ‘blood absorption index', is continuously analysed during the measurement by evaluating three spectral bands centred at 562, 576 and 593 nm, around a blood absorption peak. (**c**) The blood absorption index in relation to the relative deviation of the measured values *in vivo* from HPLC reference values *in vitro*. Data from 10 measurement sites from each of 14 subjects are shown. To guide the examiner to a site on the lower lip with an adequate blood absorption index, feedback by an indicator light is given as red, red-orange, orange and green. Measurements are only started at sites with a blood absorption index >0.7 × 10^−2^ (green) to ensure that the relationship between the detected red blood cell ZnPP fluorescence and the ZnPP/haem ratio is preserved.

The molar ratio of zinc protoporphyrin to haem, or μmol zinc protoporphyrin per mol haem, is the preferred unit for measurement of red blood cell zinc protoporphyrin^22^. With the conventional haematofluorometer, the amplitude of the red blood cell zinc protoporphyrin fluorescence is proportional to the molar *ratio* of the concentration of zinc protoporphyrin to the concentration of haemoglobin rather than to the concentration of zinc protoporphyrin alone^23^ (see Supplementary Note 1 for derivation). Similarly, under the conditions of our fibre-based measurements, the detected zinc protoporphyrin fluorescence is proportional to the zinc protoporphyrin/haem ratio and, over a wide range (Fig. 3a), nearly independent of the red blood cell concentration. In our measurements of the wet vermillion of the lower lip, the proportionality of zinc protoporphyrin fluorescence to the zinc protoporphyrin/haem ratio is preserved at sites that can be identified in real time by the magnitude of the decrease in the tissue background fluorescence spectrum resulting from blood absorption (Fig. 3b), termed hereafter the tissue ‘blood absorption index'.

For a constant zinc protoporphyrin/haem ratio, Monte Carlo simulations indicated that deviations of the detected zinc protoporphyrin fluorescence are found for variations in epithelial thickness, in light scattering coefficient, in the amount of blood present in the tissue volume, termed the tissue blood volume fraction, and in the average blood vessel diameter (Supplementary Fig. 5a–d for Monte Carlo simulation results). The influence of light scattering is virtually eliminated for a broad range of possible tissue parameters by the appropriate choice of the diameter of the excitation and detection fibre, in our case 1,000 μm (Supplementary Fig. 5c), which also leads to a high amount of detected fluorescence photons (Supplementary Fig. 5e; see Supplementary Note 1 for details about the choice of the fibre diameter). For other mucosal tissue properties, including the red blood cell concentration, the blood volume fraction, the mean blood vessel diameter, epithelial thickness and other factors, we found that the blood absorption index (Fig. 3b) correlates with the deviation of the detected zinc protoporphyrin fluorescence *in vivo* from reference HPLC measurements *in vitro*. A comparison of tissue fluorescence measurements on 14 subjects in a preparatory study with the corresponding HPLC determinations is shown in Fig. 3c (see also Supplementary Fig. 6a,b for a comparison with Monte Carlo simulations). In non-invasive measurements, the blood absorption index is used to prospectively identify suitable tissue sites for quantitative measurements, that is, those sites where only minor deviations of the detected zinc protoporphyrin fluorescence from reference HPLC values are expected. In our instrument, we determine the blood absorption index in real time. Active feedback in the form of a series of LED indicators (black box in Fig. 1b) is given to the examiner as the fibre-optic probe is moved over the lower lip. When the fibre-optic probe reaches an area with a blood absorption index above the pre-defined value (Fig. 3c), the examiner can activate the measurement process. Measurements were begun in the clinical study reported here only when the blood absorption index exceeded the lower threshold of 0.7 × 10^−2^. To maintain stable tissue perfusion during the measurement, the 1,000-μm fibre is embedded into a 12-mm steel ferrule (see Supplementary Fig. 2b for a photograph), reducing the pressure of the probe on the tissue.

### Evaluation in women after childbirth

**Figure 4:**
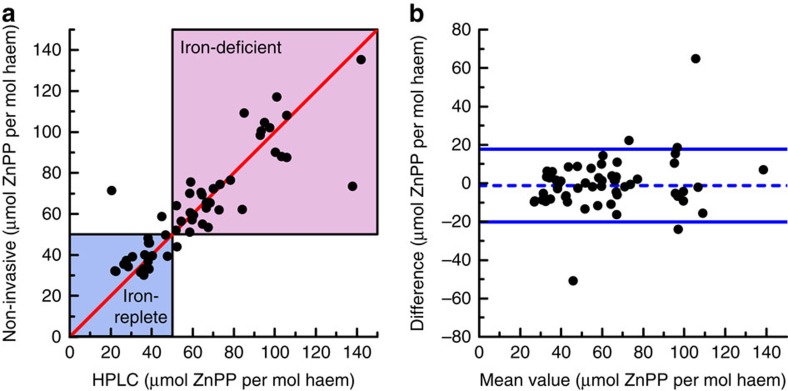
Comparison of the non-invasive measurements with the reference HPLC method in the clinical study of 56 women after childbirth. (**a**) Comparison of the scaled non-invasive measurements *in vivo* with reference HPLC measurements *in vitro*. The blue area indicates values for subjects who are iron-replete (red blood cell ZnPP<40 μmol per mol haem) or in an indeterminate zone (red blood cell ZnPP 40–50 μmol per mol haem, in which elevations do not necessarily indicate iron deficiency but may result from recent infection, inflammation or other factors). The red-shaded area indicates those who are functionally iron-deficient (red blood cell ZnPP>50 μmol per mol haem). The line of identity is shown by the diagonal line in red. (**b**) Bland–Altman plot of the data presented in **a**. The blue lines indicate the robust limits of agreement (equalling 1.96 times the robust s.d.). The bias is indicated by the dashed blue line. Details for the two outliers are provided in the Supplementary Note 2.

The performance of the non-invasive method for detection of iron deficiency was evaluated in a clinical study of 56 women within 3 days after childbirth. The mean haemoglobin concentration after delivery was 10.9 g dl^−1^ (range: 6.7–15.8 g dl^−1^). The correlation between the averaged non-invasive measurements *in vivo* and the mean of triplicate reference HPLC measurements *in vitro* is shown in Fig. 4a (Spearman's rho *r*_s_=0.87). The robust limits of agreement between the non-invasive and reference HPLC measurements, shown in the Bland–Altman plot in Fig. 4b, were 19 μmol per mol haem (95% confidence interval (CI): 14–24 μmol per mol haem, bias: −1 μmol per mol haem). For comparison, the robust limits of agreement between separate determinations by the HPLC method were 16 μmol per mol haem (95% CI: 13–19 μmol per mol haem, bias: 4 μmol per mol haem; see Supplementary Fig. 7 for the correlation and a Bland–Altman plot). We used the HPLC measurements to classify subjects as functionally iron-deficient (zinc protoporphyrin>50 μmol mol^−1^ haem) or as iron-replete or indeterminate (zinc protoporphyrin≤50 μmol per mol haem; Fig. 4). For the non-invasive method, the sensitivity for detection of iron deficiency was 97% (95% CI: 87–100%) with a specificity of 90% (95% CI: 73–98%), positive predictive value of 94% (95% CI: 85–98%) and a negative predictive value of 95% (95% CI: 79–99%).

The precision of the non-invasive measurements, determined as the s.e.m. from the 100 zinc protoporphyrin amplitudes measured for each subject, was 1.9 μmol per mol haem (range: 1.1–2.9 μmol per mol haem, see Supplementary Fig. 8 for the distribution). Interexaminer variability was studied by five examiners who were trained in use of the optical fibre for 5 min. Then, each examiner conducted measurements on the same subject. The s.d. of the average zinc protoporphyrin values was 4 μmol per mol haem for these five examiners. The repeatability of the non-invasive tissue measurements was evaluated by comparing the average over the first 50 zinc protoporphyrin amplitudes acquired from five different tissue locations, with the last 50 from the other five tissue locations for all 56 subjects. The limits of agreement of the difference of the corresponding average values were 15 μmol per mol haem (1.96 × s.d.).

Following the usual practice in field studies, unwashed whole-blood samples from the 56 subjects were also measured using a conventional haematofluorometer. We used receiver operating characteristic analysis to determine an optimal threshold of >115 μmol per mol haem to classify subjects as functionally iron-deficient according to the HPLC results. The corresponding sensitivity for detection of iron deficiency was 89% (95% CI: 76–96%), with a specificity of 90% (95% CI: 73–98%), positive predictive value of 94% (95% CI: 84–98%) and a negative predictive value of 83% (95% CI: 68–91%).

## Discussion

These results provide proof-of-concept validation for non-invasive detection of iron deficiency by optical fibre probe fluorescence spectroscopy of the lower lip. Close quantitative agreement was found between the results of measurements of red blood cell zinc protoporphyrin by the fluorescence method *in vivo* and by a standard HPLC reference assay in blood samples *in vitro* (Fig. 4a,b). In this population of women examined shortly after childbirth, the optical measurements in the lower lip had a sensitivity of 97% and a specificity of 90% for iron deficiency (Fig. 4a), using zinc protoporphyrin/haem ratios >50 μmol per mol haem as determined by HPLC as the criterion for functional iron deficiency. These fluorescence measurements were made possible by the integration of three technical innovations in the portable instrumentation reported here. First, spectral analysis of the effect of haem absorption on the tissue background fluorescence spectrum identifies suitable tissue sites, with a sufficiently thin epithelial layer and an adequate amount of blood in the tissue volume examined (Fig. 3). Second, the dual-wavelength excitation method reduces the contribution of the background fluorescence in the difference spectrum by more than 90% (Fig. 1d,e). Third, spectral fitting extracts the zinc protoporphyrin component from the difference spectrum (Fig. 2). The combination of these elements in the device reported here makes possible quantitation of red blood cell zinc protoporphyrin/haem ratio in the tissue of the lower lip *in vivo* with a precision approaching that of the standard reference HPLC method in a blood sample *in vitro*. The limits of agreement of 19 μmol per mol haem for the comparison of the non-invasive measurements with HPLC are close to those of 16 μmol per mol haem for the comparison of the samples that were measured on two occasions by HPLC.

Remarkably, the sensitivity and specificity of our non-invasive technique are at least comparable to those of a conventional haematofluorometer (sensitivity of 89% and specificity of 90%) using a drop of unwashed whole blood, as is usually done under field conditions. Washing the red blood cells is known to improve the precision of measurements by the conventional haematofluorometer^24^, but requires additional laboratory facilities and trained personnel. As shown in a previous study, the dual-wavelength excitation method effectively reduces plasma bilirubin fluorescence^19^. The spectral fitting algorithm, by eliminating unstructured background fluorescence in the difference spectrum, likely suppresses interference from medications and other components in the plasma or bound to erythrocyte membranes, but this effect has not been systematically examined.

As yet, we have examined the non-invasive method only in adult women. We plan to extend our studies to adult men and also to children. The conditions of measurements in children are likely to be favourable physiologically, with a thinner epithelium and abundant vasculature. While perioral pigmentation of the wet vermillion of the lower lip is uncommon^25^,^26^ and no interference with the non-invasive measurements was found in six subjects with dark skin pigmentation included in our series, we have not yet determined whether measures to detect and correct for the presence of melanin are needed.

Red blood cell zinc protoporphyrin is an established indicator of iron deficiency^11^,^27^ that has been used clinically for almost 50 years (refs 15, 28, 29) and has been included in the periodic US National Health and Nutrition Examination Surveys^30^. Red blood cell zinc protoporphyrin provides a measure of functional iron deficiency, when a decreased supply of iron restricts production of haemoglobin and other iron-requiring compounds^31^. Worldwide, iron deficiency, with the iron supply restricted by an insufficient amount of iron in the body, is the principal cause of an elevated red blood cell zinc protoporphyrin. All biomarkers for iron deficiency are affected by malaria, by the inflammatory response to infection, or both, and may be influenced by conditions other than iron deficiency^31^,^32^. Red blood cell zinc protoporphyrin is more stable than other screening tests for iron deficiency, such as serum ferritin, that can fluctuate within hours. In contrast, the amount of zinc protoporphyrin in the peripheral blood changes only slowly as newly formed red blood cells replace those at the end of their lifespan of ∼3–4 months. In regions endemic for malaria and other infections, the World Health Organization recommends red blood cell zinc protoporphyrin as the preferred indicator of iron deficiency in children^13^. Increases in red blood cell zinc protoporphyrin also may be produced by conditions other than iron deficiency. These disorders include lead or other heavy metal exposure^33^,^34^, some haemoglobinopathies^35^,^36^,^37^,^38^,^39^ and a variety of uncommon or rare genetic and acquired disorders, including certain porphyrias^40^ and a variety of sideroblastic and inherited microcytic anaemias^41^. Generally, these conditions are not a barrier to the use of red blood cell zinc protoporphyrin to screen for iron deficiency, but the procedures used will need to take account of their prevalence in the specific population examined (see Supplementary Note 3 for a more detailed description of these conditions). In clinical care, referral to a physician for further evaluation may be necessary^32^.

The ultimate goal of this research is to engineer the instrumentation for reliable, virtually maintenance-free field operation. The principal component groups of the instrument consist of a light source for dual-wavelength excitation, an optical fibre probe head, a detection unit and electronics with display of results. While the proof-of-concept device reported here was assembled using commercially available parts, custom-made components could substantially reduce device size and weight. The most expensive component in the current instrument is the spectrometer. The extent by which instrumental cost could be reduced by use of less expensive photodetectors with sufficient dynamic range and spectral resolution remains to be determined. A specifically designed electronics board with a programmable microprocessor and touch panel user interface could replace the computer and the separate control electronics. Given the low-power consumption (18 W), conversion to battery operation is straightforward. With a housing specifically designed to accommodate the optics, beam steering components and electronics, we anticipate that the instrument could be configured as a robust, inexpensive unit with the dimensions of a paperback book and a weight less than 0.5 kg. This fluorescence method potentially provides a rapid, easy to use means for point-of-care screening for iron deficiency in resource-limited settings lacking laboratory infrastructure. More generally, dual-wavelength excitation with spectral fitting is potentially useful in quantifying other blood-bound fluorophores, especially in studies of the pharmacokinetics of photosensitizers in photodynamic applications.

## Methods

### Non-invasive fluorescence measurements

For fluorescence measurements, subjects sat in front of the examiner, gently turning the lower lip outwards. The examiner placed the fibre-optic probe on the wet vermillion of the subject and switched on the ‘search mode' using a foot-switch. In this mode, fluorescence spectra were acquired continuously with an integration time of 200 ms on 407-nm excitation and analysed for the absorption caused by blood at its absorption maximum ∼576 nm (Fig. 3b). The blood absorption index *a* was determined such that the decrease in the tissue-background fluorescence around 576 nm could be removed by dividing the tissue fluorescence spectrum by 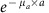 where *μ*_*a*_ is the blood absorption. If the blood absorption index exceeded the pre-defined value of 0.70 × 10^−2^ (Fig. 3c), a green light was illuminated, indicating to the examiner that the current position on the lower lip was suitable for a reliable red blood cell zinc protoporphyrin determination. For a blood absorption index between 0.42 × 10^−2^ and 0.70 × 10^−2^, a orange light was shown; between 0.14 × 10^−2^ and 0.42 × 10^−2^, red and orange lights; and below 0.14 × 10^−2^, a red light. The examiner moved the fibre-optic probe slowly over the vermillion of the subject until a green light was displayed, typically in ∼2–3 s. Then, the examiner began the measurement procedure by using a foot-switch.

During the measurement procedure, the 407- and the 425-nm excitation laser diodes were subsequently switched on and the resulting tissue fluorescence spectra were acquired with a 200-ms integration time. To exclude marked variations in tissue optical properties during this integration time, additional remission measurements were carried out at the excitation wavelengths and with a 520-nm laser diode using the surrounding fibres in the fibre bundle (Supplementary Fig. 2b). The fibre bundle was attached to a photodiode that was read out with 2,000 samples at a rate of 10 kHz. No marked variations were found, and these remission measurements do not enter into the calculation. Finally, all laser diodes were switched off, and a dark spectrum and the photodiode dark signal were collected with the above settings. This measurement cycle (successively switching the three laser diodes on, finally switching all laser diodes off) was repeated 10 times, with a total duration of 10 s. All spectra and photodiode samples were stored electronically.

During the 10-s measurement procedure, the examiner held the fibre-optic probe in place at the tissue site. Afterwards, the examiner decided whether the measurement at this site had been conducted under controlled circumstances, that is, that the subject did not move and the fibre-optic probe did not lose contact to the tissue during the measurement. In that case, the measurement was considered valid. The procedure of starting the search mode, identifying a suitable measurement site, starting the measurement and then deciding whether the measurement was carried out under controlled conditions, was repeated until 10 valid measurements per patient had been conducted. Generally, no measurement had to be excluded (on average 0.2 measurements were excluded per subject, range 0–2, with a total of 12 invalid and 560 valid measurements). Typically, the whole measurement procedure took less than 5 min. The total net measurement time was ∼100 s (10 times 10 s).

### Tissue data evaluation and spectral fitting

The acquired tissue fluorescence spectra were evaluated using MATLAB (version R2013a, The MathWorks Inc., Natick, MA, USA). After the measurement for each subject, totals of 100 spectra excited at 425 nm, 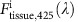, 100 excited at 407 nm, 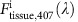 and 100 dark spectra 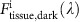 were available. First, from each fluorescence spectrum, the corresponding dark spectrum was subtracted. Then, the resulting spectrum was divided by the integration time, 200 ms, normalizing the spectra to 1 ms, yielding 100 calibrated tissue spectra for each excitation wavelength, 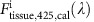 and 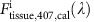. ‘Difference spectra' 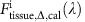 were calculated from the calibrated fluorescence spectra by normalizing 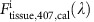 to 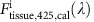 in the wavelength range 520–525 nm and subsequent subtraction. In these difference spectra, spectral features of blood absorption *A*_blood_(*λ*) (Supplementary Fig. 3), zinc protoporphyrin fluorescence *F*_ZnPP_(*λ*) and protoporphyrin IX fluorescence *F*_PPIX_(*λ*) (Supplementary Fig. 4) are present (Fig. 2a). These spectral features were removed from the difference spectra 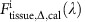 by optimizing the fit parameters *a*, *z* and *p* so that the remaining fluorescence background was smooth in the wavelength range 560–750 nm (Fig. 2c). This is the case when the second derivative of the background with spectral features removed is as close to zero as possible (Fig. 2b). This procedure is illustrated by the following equation:


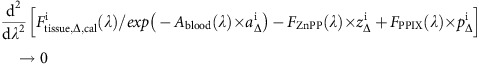


Finally, the zinc protoporphyrin value for each subject was calculated by averaging over the 100 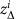 measured for each subject. These values are given in Supplementary Table 1.

### HPLC

HPLC was performed at the Deutsches Kompetenz-Zentrum für Porphyriediagnostik und Konsultation, MVZ Labor PD Dr. Volkmann und Kollegen GbR, Karlsruhe, Germany, using a system consisting of a Waters autosampler 717 with pump 515, and a Shimadzu RF 20-AL detector. A Knauer Nucleosil C18 column (250 × 4.6 mm, solvent 56% acetone, 24% methanol, 20% water and 0.02% formic acid (v/v/v)) was operated with a flow rate of 2.0 ml min^−1^. Fluorescence was excited at 417 nm and detected at 635 nm (20 nm spectral bandwidth). A calibration standard (KC2700KA, Immundiagnostik AG, Bensheim, Germany) and two controls were measured together with the samples. EDTA-anticoagulated blood samples from all 56 study subjects were stored at −80 °C and were measured in one batch. After defrosting, the samples were homogenized on a multiaxle rotating mixer for at least 3 min. Then, 30 μl of the sample together with 100 μl haemolysis solution (96% water and 4% formic acid) and 1,000 μl acetone were vortexed for 30 s and subsequently centrifuged at 10,000 r.p.m. Fifty microlitres of the supernatant were then injected into the HPLC system. For calculation of the zinc protoporphyrin/haem ratio, the haemoglobin concentration was determined from the same aliquots using a Sysmex XE-2100 haematology analyser (Sysmex Corporation, Kobe, Japan) using the SLS (cyanide-free sodium lauryl sulphate) method.

To minimize intra-assay variations, for each subject, three samples were prepared according to this protocol and the zinc protoporphyrin and the protoporphyrin IX concentrations determined. For comparison with the other methods, the mean values of these triplicate measurements were used. In addition, inter-assay variations were assessed by measuring aliquots of all 56 subjects in repeated batches. These batches are compared in a Bland–Altman plot (see Supplementary Fig. 7).

### Clinical evaluation of non-invasive optical measurements

Clinical evaluation of the non-invasive instrument was carried out in the Perinatal Center, Klinikum der Universität München, Munich, Germany. A series of studies was conducted in three phases, with 20 subjects in each of the first two preparatory phases and 56 subjects in the last phase. For participation in the study, exclusion criteria were (i) transfusion of blood products in the course of delivery, (ii) a diagnosis of thalassaemia or sickle cell anaemia and (iii) any acute or chronic infectious or inflammatory disease. Between each phase of the clinical studies, instrumental improvements were made. We focus here on the results of the last phase, with a recruitment period from March 2014 to December 2014. For estimation of the CIs of the limits of agreement between the non-invasive and reference HPLC methods, the sample size of 56 provided a 95% CI of about ±0.45*s*, where *s* is the s.d. of the differences between measurements by the two methods^42^. Ethical approval for the study was given by the Institutional Ethical Board of the Klinikum der Universität München (study identifier: LFL_01/2012, ClinicalTrials.gov identifier: NCT02310607). Written informed consent was obtained from each subject.

All non-invasive fluorescence measurements were conducted by the same physician during the clinically indicated inpatient stay within 3 days after delivery. For each subject, 10 non-invasive measurements at each of 10 different sites on the wet vermillion of the lower lip were performed. The fit amplitudes for the red blood cell zinc protoporphyrin were averaged for each subject and scaled to the HPLC value range for comparison. The results were compared with the zinc protoporphyrin/haem ratio measured by the reference HPLC method from whole blood for each subject, as described below. After the measurements, study participants had a medical consultation to discuss the results of the iron deficiency parameters measured from the blood samples.

Aliquots from residual EDTA-anticoagulated blood samples obtained after clinically indicated blood withdrawal were anonymized. Two aliquots of 500 μl each were frozen at −80 °C and were used for zinc protoporphyrin determination by HPLC at the Deutsches Kompetenz-Zentrum für Porphyriediagnostik und Konsultation, MVZ Labor PD Dr. Volkmann und Kollegen GbR. A 200-μl aliquot was stored at 8 °C for at most 3 days, and used for determination of the red blood cell zinc protoporphyrin/haem ratio with a commercial haematofluorometer (Model 206D, AVIV Biomedical Inc., Lakewood, NJ, USA) according to the manufacturer's instructions, using materials for calibration and quality control obtained from the manufacturer.

### Statistical data analysis

The zinc protoporphyrin values measured by the non-invasive fluorescence method yielded arbitrary units. To directly compare these units with HPLC values, the zinc protoporphyrin values were scaled. The scaling factor was the slope of a straight line with zero offset fitted to the comparison of the zinc protoporphyrin values with the mean HPLC values of the triplicate measurements (software: R version 3.2.0, function: *lmrob*, package: *robustbase* version 0.92-3). Bland–Altman plots show the difference of the scaled zinc protoporphyrin values and the mean of the HPLC triplicate measurements against the mean of both values. To compare the two methods, the limits of agreement and bias shown in the Bland–Altman plots were calculated using a robust s.d., the robust *τ*-estimate (software: R, function: *scaleTau2*, package: *robustbase*) of the differences between the two methods, multiplied by 1.96. The 95% CIs were calculated by bootstrapping (software: R, functions: *boot* and *boot.ci*, package: *boot* version 1.3-16). The optimal threshold for the haematofluorometer measurements was determined by receiver operating characteristic analysis against classification by HPLC (zinc protoporphyrin >50 μmol per mol haem; software: R, function: *optimal.cutpoints*, package: *OptimalCutpoints* version 1.1-3). Sensitivity and specificity were calculated from contingency tables (software: R, function: *BDtest*, package: *bdpv* version 1.1).

### Monte Carlo simulations

To study the influence of tissue geometry, tissue optical parameters, as well as the geometry of the fibre-optic probe, Monte Carlo simulations were performed. These simulations were conducted using a modified version of a graphic card implementation of the simulation programme ‘Monte Carlo Multi-Layered' (MCML)^43^,^44^. The modifications include implementation of fluorescence simulation using the weighted direct emission method^45^ and simulation of optical fibres. For this modified MCML programme, photons are injected at the position of an optical fibre at the tissue surface, equally distributed across the fibre diameter, while the initial angle of the photon is chosen according to a cosine distribution but limited by the numerical aperture (NA) of the optical fibre. For all simulations, the NA of the optical fibres in air was NA=0.22 and their refraction index *n*_fibre_=1.46. The refraction index of the tissue was chosen as *n*_tissue_=1.33. The simulated tissue consists of multiple tissue layers with finite thickness and infinite extent in the plane. The photons injected by the excitation fibre propagate through the layers, with each layer having a defined absorption coefficient *μ*_a,exc_, scattering coefficient *μ*_s,exc_ and anisotropy parameter *g*_exc_ for the Henyey–Greenstein phase function. The fluorescence photons generated are emitted isotropically and tracked until they leave the tissue, with optical parameters *μ*_a,em_, *μ*_s,em_ and *g*_em_ defined for each layer. Both excitation and fluorescence photons were cancelled in case their weight became too small by ‘survival roulette', while conservation of energy was ensured^44^,^45^,^46^. The photons detected by one or more optical fibres, including the excitation fibre, are recorded, summarized and normalized to the number of excitation photons injected.

The simulated tissue consisted of three layers, the epithelium, the superficial stroma and the lower stroma. The properties of the superficial and the lower stroma differed only by the blood content. The optical properties of the epithelium and the stroma were varied to ensure that the physiological range is covered (see Supplementary Tables 2 and 3). For Supplementary Fig. 5a, additionally a blood content of 16% in the lower stroma was simulated. The epithelium thickness was also varied (50, 100, 200 and 400 μm) for each combination of optical properties in Supplementary Tables 2 and 3. The superficial stroma was 200-μm thick, while the thickness of the lower stroma was set to 10 mm. Fluorescence was generated at the emission wavelengths of 561 and 576 nm (tissue autofluorescence only) and 593 nm (tissue autofluorescence and zinc protoporphyrin fluorescence). Zinc protoporphyrin fluorescence was generated only in the blood-perfused stroma. The amount of fluorescence photons generated was dependent on the respective absorption coefficient. The contribution of the fluorophores to the respective absorption coefficient was chosen as 0.1. The quantum efficiency was chosen as *γ*=1. Fluorescence photons detected by optical fibres were stored separately for the different tissue layers and fluorophores. In addition, the relative number of detected excitation photons was recorded. The total number of excitation photons was 10^8^ for each simulation.

To study the influence of different average blood vessel diameters in the examined tissue volume, the blood absorption as input parameter for the simulations was adapted. An average blood vessel diameter of 24 μm (s.d.: 14 μm) was found by Amelink *et al*. for normal oral mucosa^47^. Therefore, in addition to evenly distributed erythrocytes, blood vessel diameters of 24, 38 and 52 μm were simulated to cover the physiological range. Larger blood vessels reduced the blood absorption, especially at wavelengths with high blood absorption according to the formula described by van Veen *et al*.^48^. The resulting absorption coefficient of 1% blood at 425 nm was then 0.413, 0.263 and 0.192 mm^−1^ for 24, 38 and 52 μm vessel diameters, respectively; at 561 nm it was 0.153, 0.136 and 0.121 mm^−1^; at 576 nm it was 0.206, 0.174 and 0.148 mm^−1^; and at 593 nm it was 0.056, 0.054 and 0.052 mm^−1^.

The absorption caused by blood at its absorption maximum around 576 nm, the blood absorption index, was quantified by using the autofluorescence detected at 561 and 576 nm. Both detected autofluorescence signals were divided by 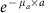, with *μ*_*a*_ the respective blood absorption coefficient (Supplementary Fig. 3), where the blood absorption index *a* was varied such that the result was equal for both wavelengths.

To study the influence of the fibre diameter, separate simulations with optical fibres with diameters 200, 400, 600, 800, 1,000 and 1,500 μm were performed, with the fibres embedded in a perfectly reflecting 12-mm ferrule. For the other simulations, the fibre-optic probe shown in Supplementary Fig. 2b was simulated.

For comparison with the fluorescence detected from a whole-blood sample, the following Monte Carlo simulation was performed. The fibre-optic probe was in contact with a 1-cm-thick whole-blood sample. The blood was assumed to contain 96% oxygenized and 4% deoxygenized haemoglobin. Packaging was not considered because of the high absorber concentration. The absorption of the sample was therefore 188.49 mm^−1^ (excitation wavelength, 425 nm) and 4.79 mm^−1^ (fluorescence wavelength, 593 nm), respectively. Scattering of the whole-blood sample was disregarded.

## Additional information

**How to cite this article:** Hennig, G. *et al*. Non-invasive detection of iron deficiency by fluorescence measurement of erythrocyte zinc protoporphyrin in the lip. *Nat. Commun.* 7:10776 doi: 10.1038/ncomms10776 (2016).

## Supplementary Material

Supplementary Information Supplementary Figures 1-8, Supplementary Tables 1-3, Supplementary Notes 1-3 and Supplementary References
