# Supplementary material for: Non-invasive detection of iron deficiency by fluorescence measurement of erythrocyte zinc protoporphyrin in the lip
Source: Nat Commun. 2016 Feb 17;7:10776. doi: 10.1038/ncomms10776 (PMC4757790; doi:10.1038/ncomms10776)
Supplement: Supplementary Information — Supplementary Figures 1-8, Supplementary Tables 1-3, Supplementary Notes 1-3 and Supplementary References [file ncomms10776-s1.pdf]

SUPPLEMENTARY FIGURES

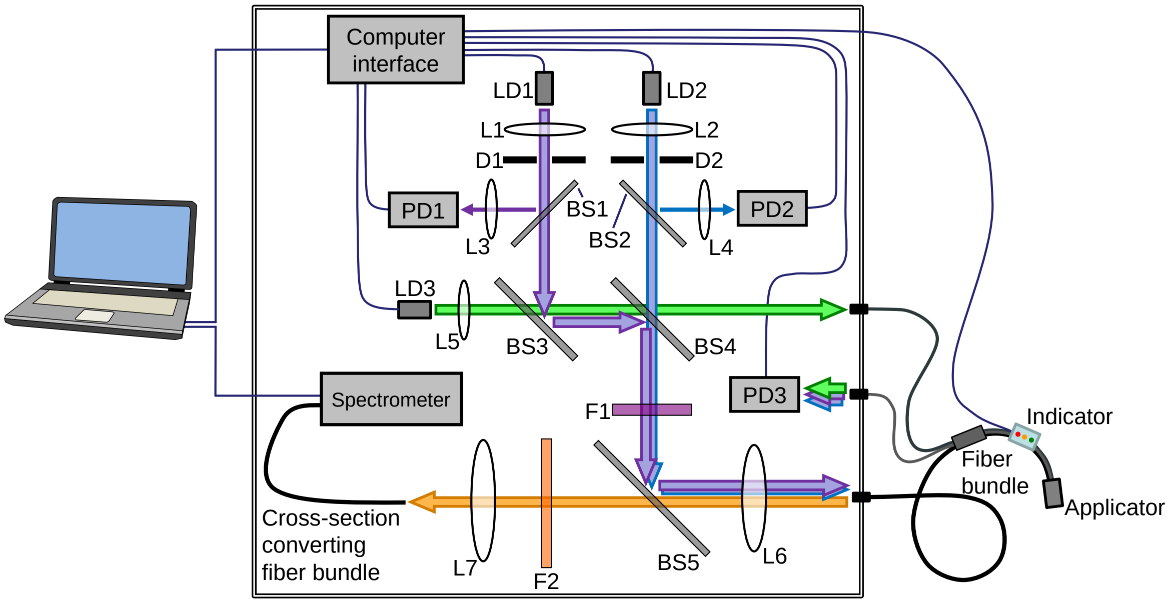

Supplementary Figure 1. Schematic of the set-up. Light from the 407 nm laser diode LD1 (20 mW, central wavelength: 407.0 nm, bandwidth: 1.9 nm full width at half maximum, FWHM) and from the 425 nm laser diode LD2 (50 mW, central wavelength: 424.7 nm, bandwidth: 2.1 nm FWHM) is collimated by lens L1 and L2, respectively. The light output power is restricted by the iris diaphragms D1 and D2 to 2.5 mW as measured by a calibrated power meter (LM-2 VIS sensor head, connected to FieldMaster, Coherent Inc., Santa Clara, CA, USA). Part of each beam is coupled out by the beam splitters BS1 and BS2 and loosely focused by lenses L3 and L4 onto 9 mm^2 silicon photodiodes PD1 and PD2, which are used to monitor the intensity of the laser diodes. The two beams are superimposed by the dichroic beam splitters BS3 and BS4. Band-pass filter F1 is introduced to remove parasitic light in the spectral range of zinc protoporphyrin fluorescence. The excitation light is reflected by dichroic beam splitter BS5 and coupled by lens L6 into a 1000 µm fused silica fiber (numerical aperture (NA) = 0.22) that is part of the fiber bundle. The end of the fiber bundle is embedded in a stainless steel ferrule with a diameter of 12 mm that is brought in contact with the wet vermillion of the subject. Fluorescence light from the tissue is collected with the same 1000 µm fiber, is collimated by L6, passes through BS5, is spectrally filtered by the long-pass filter F2 (GG495, Schott AG, Mainz, Germany), and is coupled by lens L7 into a

cross-section converting fiber bundle (7x 200 µm fibers, round to linear, NA = 0.22, LightGuideOptics Germany GmbH, Rheinbach, Germany), which is connected to the spectrometer (cooled CCD spectrometer, detector size 24.6 mm x 1.4 mm, 198-986 nm, 1044 pixels, QE65Pro-FL, OceanOptics, Inc., Dunedin, FL, USA). In addition to the excitation light at 407 nm and 425 nm, green light around 520 nm from a third laser diode LD3 (5 mW, restricted to 0.8 mW, quasi-continuous-wave, central wavelength: 518.9 nm, bandwidth: 2.0 nm FWHM) is coupled by lens L5 into a fiber bundle (5 fibers, 400 µm diameter each, NA = 0.22), which surrounds the 1000 µm fiber used for fluorescence excitation and detection (Supplementary Fig. 2*b*). A second fiber bundle (5 fibers, 400 µm diameter each, NA = 0.22) is used to collect light remitted from the tissue surface at 407 nm, 425 nm and 520 nm. This remitted light is detected by photodiode PD3. An LED indicator (red, orange, green) is attached to the fiber bundle, which is used to give optical feedback about the blood absorption index to the examiner. Photographs of the device and the fiber-optic probe tip are shown in Supplementary Figure 2. The laser diodes, photodiodes and the spectrometer are controlled by a laptop running a custom program (LabVIEW version 8.5, National Instruments Corporation, Austin, TX, USA).

a **b**

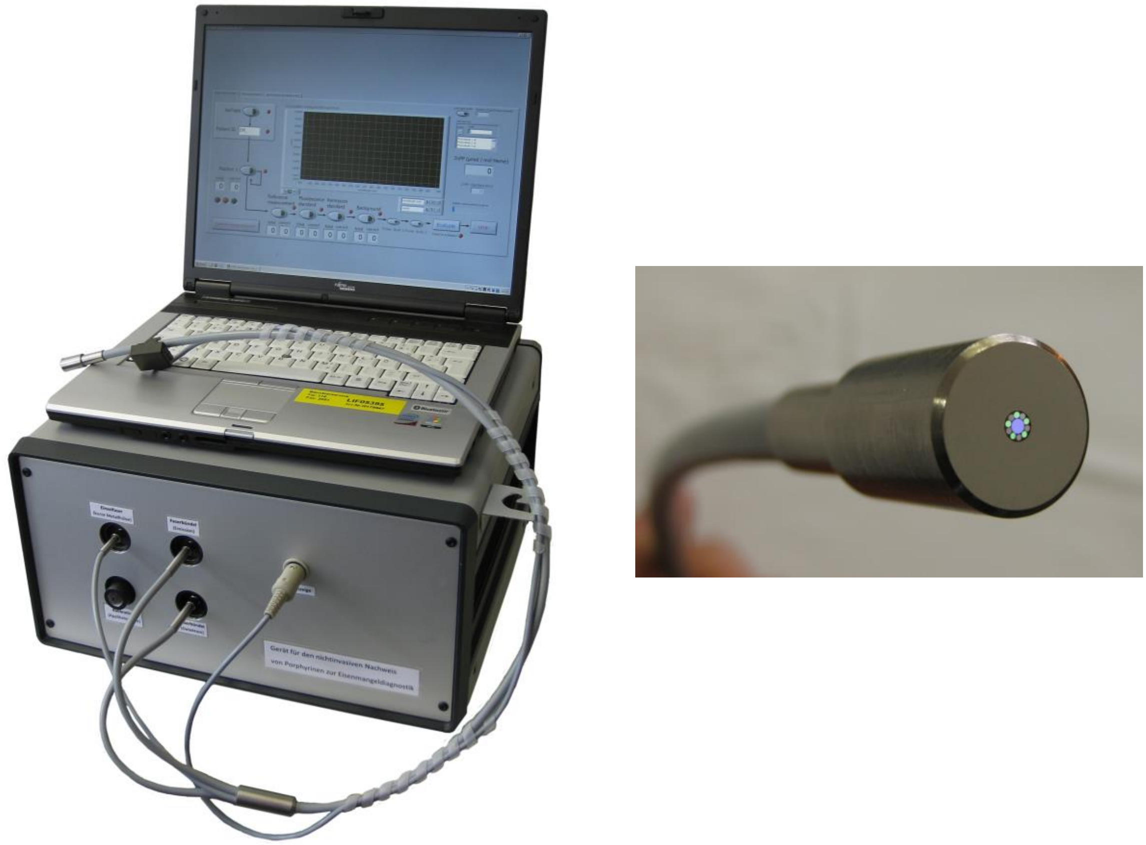

Supplementary Figure 2. Photographs of the instrument. (a) Photograph of the instrument with fiber-optic probe attached and the laptop used for controlling the instrument. **(b)** Photograph of the fiber-optic probe tip. The 1000 µm excitation and fluorescence detection fiber (center, colored blue) is surrounded by ten 400 µm fibers, of which five are used for the detection of remitted light (colored grey) and five for delivery of green light for remission measurements (colored green). The optical fibers are embedded in a 12 mm stainless steel ferrule with chamfered edge.

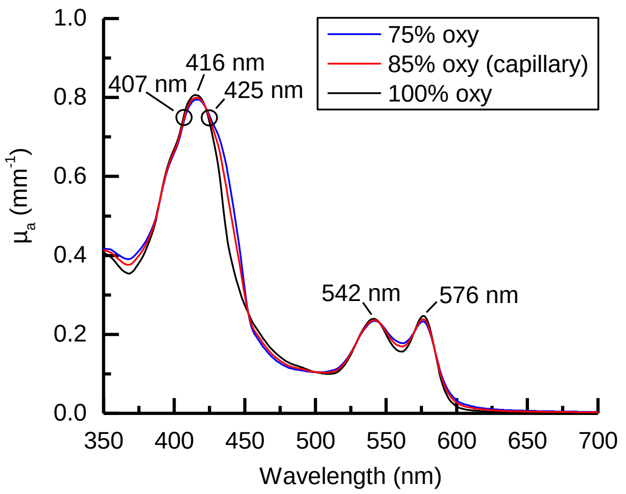

Supplementary Figure 3. Blood absorption spectra for the range of physiologically relevant oxygenation states. The capillary blood absorption spectrum (85% oxygen saturation) is used for computer simulations and spectral fitting. Indicated are the predominant absorption maxima, as well as the two excitation wavelengths used for the non-invasive measurements. The spectra were calculated using the compiled oxygenized and deoxygenized hemoglobin absorption data by Scott Prahl[1] with a hemoglobin concentration of 15 g dl^{-1}. The packaging effect of hemoglobin found in erythrocytes at a low concentration of erythrocytes was taken into account,[2] using a hematocrit of 41% and red blood cell size of 7 µm. The absorption spectra are calculated for 1% blood concentration. For the computer simulations, the capillary blood absorption spectrum was scaled for different blood concentrations.

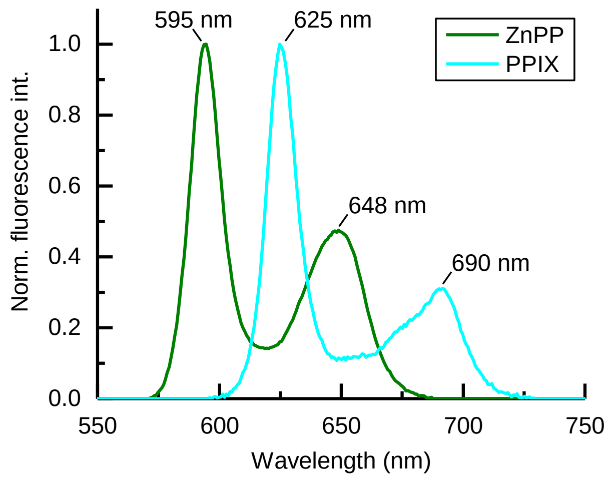

Supplementary Figure 4. Fluorescence spectra. Normalized fluorescence spectra of zinc protoporphyrin (ZnPP) with 425 nm excitation and of protoporphyrin IX (PPIX) with 407 nm excitation measured on whole blood.

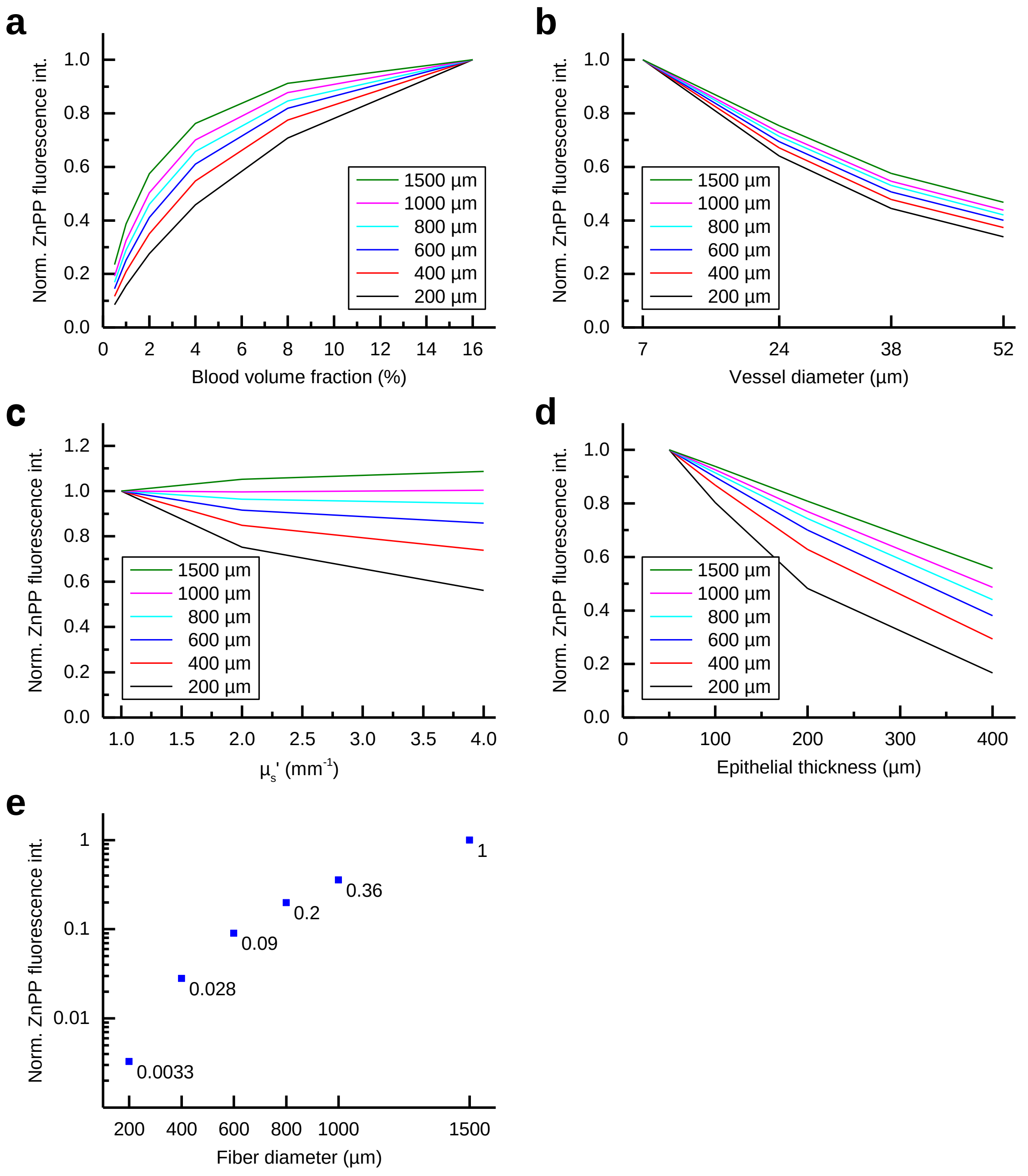

Supplementary Figure 5. Monte Carlo simulation results. Monte Carlo simulation results are shown for different optical fiber diameters (NA = 0.22) to detect zinc protoporphyrin fluorescence from tissue (for the optical tissue parameters used, see Supplementary Tables 2 and 3). The normalized fluorescence is plotted as a function of **(a)** a varying blood volume fraction (BVF) for evenly distributed erythrocytes within the sample volume, the reduced scattering coefficient $\mu_s' = 2.0\ mm^{-1}$ and epithelial thickness 200 µm, **(b)** a varying mean blood vessel diameter in the sample volume (for 7 µm, the erythrocytes were distributed

evenly within the sample volume) with BVF = 4.0%, μ_s' = 2.0 mm^{-1} and epithelial thickness 200 µm, **(c)** a varying μ_s' for evenly distributed erythrocytes within the sample volume, BVF = 4.0% and epithelial thickness 200 µm, **(d)** a varying epithelial thickness for evenly distributed erythrocytes within the sample volume, BVF = 4.0% and μ_s' = 2.0 mm^{-1}, and **(e)** a varying fiber diameter for evenly distributed erythrocytes within the sample volume, BVF = 4.0%, μ_s' = 2.0 mm^{-1} and epithelial thickness 200 µm. In (a)-(d), different colors shown in the legends indicate the simulated fiber diameters. In (e), the numbers indicate the normalized detected zinc protoporphyrin fluorescence value for each fiber diameter.

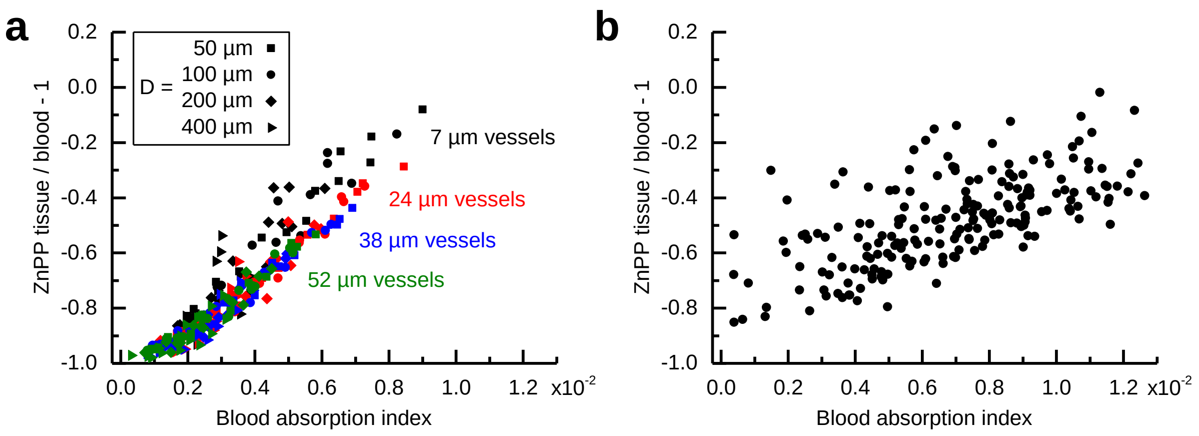

Supplementary Figure 6. Comparison of Monte Carlo simulations and tissue measurements. Relative deviation of the zinc protoporphyrin (ZnPP) fluorescence measured in tissue compared to whole blood ("ZnPP tissue/blood – 1", y-axis) plotted against the strength of the blood absorption found in the tissue background fluorescence ("blood absorption index", x-axis). To acquire fluorescence spectra in whole blood, an optical bare fiber (diameter 1000 µm) was attached to the ZnPP fluorometer and was placed in a 50 µl EDTA-anticoagulated aliquot of whole blood. In panel **(a)**, Monte Carlo simulation results are shown. Different symbols denote the epithelial thickness *D* in the range 50 µm to 400 µm, different colors the assumed average blood vessel diameter (for 7 µm, the erythrocytes were distributed evenly within the sample volume). A detailed list of optical parameters used is given in Supplementary Table 2 and 3. In **(b)**, experimental results from 14 subjects are shown. For each subject, measurements were done at 10 different tissue positions. For each position, 10 measurements were averaged, yielding the data points shown.

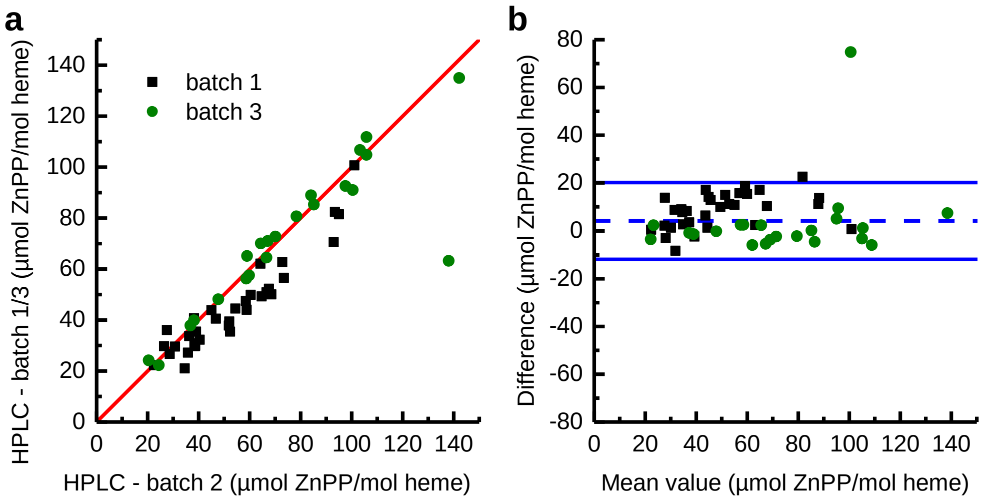

Supplementary Figure 7. Comparison of repeated HPLC measurements. Comparison of 56 samples that were measured on two occasions by the HPLC reference method in different batches on different days. Batch 2 samples were all measured on the same day and are used in Figure 4. Batch 1 (34 samples) and batch 3 (22 samples) were measured on different days to assess the reproducibility of the HPLC measurements. **(a)** Correlation of batch 2 with batch 1 and batch 3. **(b)** Bland-Altman plot showing the difference of the measurements against their mean value. Limits of agreement are 16 µmol/mol heme (95% CI: 13 to 19 µmol/mol heme, bias: 4 µmol/mol heme).

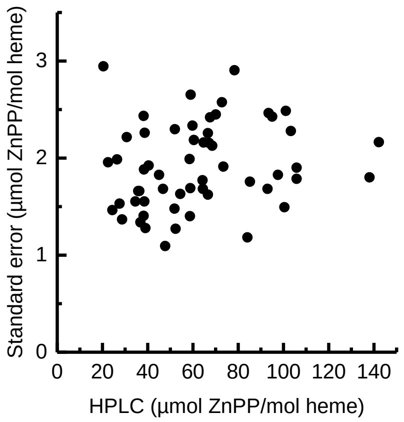

Supplementary Figure 8. Standard errors of tissue measurements. Standard errors of the 100 single non-invasive measurements for the 56 patients plotted against the corresponding HPLC value (average: 1.9 µmol/mol heme, range: 1.1 to 2.9 µmol/mol heme). The standard errors were calculated as standard deviation divided by the square root of the number of single measurements.

SUPPLEMENTARY TABLES

Supplementary Table 1. Summary of erythrocyte zinc protoporphyrin measurements. Zinc protoporphyrin values for all subjects measured by the different methods, reference HPLC, non-invasive fluorometer measurements on tissue and conventional AVIV hematofluorometer.

#	HPLC (µmol/mol heme)	ZnPP fluorometer (µmol/mol heme)	AVIV (µmol/mol heme)
1	45.0	58.5	134
2	27.7	37.3	95
3	38.2	47.9	111
4	30.8	39.1	83
5	64.3	70.4	116
6	52.3	43.8	92
7	22.6	31.8	63
8	26.5	35.3	93
9	28.7	34.1	85
10	58.5	51.0	130
11	35.9	33.1	92
12	36.3	30.0	108
13	39.0	45.7	89
14	40.4	39.5	90
15	72.9	62.0	149
16	67.6	53.4	139
17	54.5	56.4	129
18	64.8	54.9	90
19	34.6	31.4	68
20	38.7	32.9	85
21	66.7	65.3	98
22	68.6	65.3	151
23	46.9	49.5	112
24	38.5	39.0	92
25	60.5	59.3	121
26	52.1	63.9	117

#	HPLC (µmol/mol heme)	ZnPP fluorometer (µmol/mol heme)	AVIV (µmol/mol heme)
27	38.6	45.8	83
28	93.0	98.4	220
29	93.4	100.4	202
30	101.2	116.9	264
31	58.9	60.6	131
32	52.0	51.9	132
33	95.0	104.5	204
34	73.5	74.3	189
35	84.1	62.1	201
36	67.1	65.6	210
37	78.4	76.4	191
38	97.6	101.9	202
39	103.4	88.0	201
40	70.1	72.1	146
41	59.8	57.0	156
42	58.7	69.9	119
43	59.0	75.5	187
44	20.5	71.4	137
45	138.1	73.3	175
46	105.8	108.1	227
47	142.2	135.3	299
48	47.8	39.3	100
49	100.4	90.0	187
50	105.9	87.4	189
51	64.5	69.2	135
52	66.7	62.9	99
53	38.3	36.8	78
54	85.2	109.2	150
55	36.8	39.8	91
56	24.4	32.0	65

Supplementary Table 2. Optical tissue parameters of the epithelium used for the Monte Carlo simulations. The anisotropy parameter was set to g = 0.9. For each of the three combinations of optical parameters for the epithelium, five different blood volume fractions in the stroma were simulated, yielding a total of 15 simulations.

#	$\mu_{a,exc,425}$ (mm^{-1})	$\mu_{a,em,561}$ = $\mu_{a,em,576}$ (mm^{-1})	$\mu_{a,em,593}$ (mm^{-1})	$\mu_{s,exc,425}$ (mm^{-1})	$\mu_{s,em,561}$ = $\mu_{s,em,576}$ (mm^{-1})	$\mu_{s,em,593}$ (mm^{-1})
1 to 5	0.3	0.15	0.14	5.0	3.5	3.4
6 to 10	0.3	0.15	0.14	10.0	7.0	6.8
11 to 15	0.3	0.15	0.14	20.0	14.0	13.6

Supplementary Table 3. Optical tissue parameters (rounded) of the lower stroma used for the Monte Carlo simulations. The anisotropy parameter was set to g = 0.9. For the superficial stroma, a lower blood volume fraction (BVF) was assumed, reducing the blood absorption coefficients $\mu_{a,exc,425,blood}$, $\mu_{a,em,561,blood}$, $\mu_{a,em,576,blood}$ and $\mu_{a,em,593,blood}$ by 50%, thus reducing the total absorption coefficients. For each of the 15 different combinations of stromal optical parameters, the corresponding set of epithelial optical parameters was used (Supplementary Table 2).

#	BVF (%)	$\mu_{a,exc,425,blood}$ + $\mu_{a,exc,425,tissue}$ = $\boldsymbol{\mu_{a,exc,425}}$ (mm^{-1})	$\mu_{a,em,561,blood}$ + $\mu_{a,em,561,tissue}$ = $\boldsymbol{\mu_{a,em,561}}$ (mm^{-1})	$\mu_{a,em,576,blood}$ + $\mu_{a,em,576,tissue}$ = $\boldsymbol{\mu_{a,em,576}}$ (mm^{-1})	$\mu_{a,em,593,blood}$ + $\mu_{a,em,593,tissue}$ = $\boldsymbol{\mu_{a,em,593}}$ (mm^{-1})	$\boldsymbol{\mu_{s,exc,425}}$ (mm^{-1})	$\boldsymbol{\mu_{s,em,561}}$ = $\boldsymbol{\mu_{s,em,576}}$ (mm^{-1})	$\boldsymbol{\mu_{s,em,593}}$ (mm^{-1})
1	**0.5**	0.374 + 0.630 = **1.00**	0.085 + 0.160 = **0.245**	0.119 + 0.160 = **0.280**	0.029 + 0.146 = **0.175**	**10.0**	**7.0**	**6.8**
2	**1.0**	0.748 + 0.630 = **1.38**	0.169 + 0.160 = **0.330**	0.239 + 0.160 = **0.399**	0.058 + 0.146 = **0.204**	**10.0**	**7.0**	**6.8**
3	**2.0**	1.50 + 0.630 = **2.13**	0.338 + 0.160 = **0.499**	0.477 + 0.160 = **0.637**	0.116 + 0.146 = **0.261**	**10.0**	**7.0**	**6.8**
4	**4.0**	2.99 + 0.630 = **3.62**	0.677 + 0.160 = **0.837**	0.954 + 0.160 = **1.11**	0.231 + 0.146 = **0.377**	**10.0**	**7.0**	**6.8**
5	**8.0**	5.98 + 0.630 = **6.61**	1.35 + 0.160 = **1.51**	1.91 + 0.160 = **2.07**	0.462 + 0.146 = **0.608**	**10.0**	**7.0**	**6.8**
6	**0.5**	0.374 + 0.630 = **1.00**	0.085 + 0.160 = **0.245**	0.119 + 0.160 = **0.280**	0.029 + 0.146 = **0.175**	**20.0**	**14.0**	**13.6**
7	**1.0**	0.748 + 0.630 = **1.38**	0.169 + 0.160 = **0.330**	0.239 + 0.160 = **0.399**	0.058 + 0.146 = **0.204**	**20.0**	**14.0**	**13.6**
8	**2.0**	1.50 + 0.630 = **2.13**	0.338 + 0.160 = **0.499**	0.477 + 0.160 = **0.637**	0.116 + 0.146 = **0.261**	**20.0**	**14.0**	**13.6**
9	**4.0**	2.99 + 0.630 = **3.62**	0.677 + 0.160 = **0.837**	0.954 + 0.160 = **1.11**	0.231 + 0.146 = **0.377**	**20.0**	**14.0**	**13.6**
10	**8.0**	5.98 + 0.630 = **6.61**	1.35 + 0.160 = **1.51**	1.91 + 0.160 = **2.07**	0.462 + 0.146 = **0.608**	**20.0**	**14.0**	**13.6**
11	**0.5**	0.374 + 0.630 = **1.00**	0.085 + 0.160 = **0.245**	0.119 + 0.160 = **0.280**	0.029 + 0.146 = **0.175**	**40.0**	**28.0**	**27.2**
12	**1.0**	0.748 + 0.630 = **1.38**	0.169 + 0.160 = **0.330**	0.239 + 0.160 = **0.399**	0.058 + 0.146 = **0.204**	**40.0**	**28.0**	**27.2**
13	**2.0**	1.50 + 0.630 = **2.13**	0.338 + 0.160 = **0.499**	0.477 + 0.160 = **0.637**	0.116 + 0.146 = **0.261**	**40.0**	**28.0**	**27.2**
14	**4.0**	2.99 + 0.630 = **3.62**	0.677 + 0.160 = **0.837**	0.954 + 0.160 = **1.11**	0.231 + 0.146 = **0.377**	**40.0**	**28.0**	**27.2**
15	**8.0**	5.98 + 0.630 = **6.61**	1.35 + 0.160 = **1.51**	1.91 + 0.160 = **2.07**	0.462 + 0.146 = **0.608**	**40.0**	**28.0**	**27.2**

SUPPLEMENTARY NOTES

Supplementary Note 1. Quantitation of the zinc protoporphyrin/heme ratio by fluorescence spectroscopic measurements

Zinc protoporphyrin fluorescence is a quantitative measure of the zinc protoporphyrin/heme ratio. In the case of the conventional hematofluorometer, the detected red blood cell zinc protoporphyrin fluorescence is proportional to the molar *ratio* of the concentration of zinc protoporphyrin to the concentration of hemoglobin rather than to the concentration of zinc protoporphyrin alone.[3] This relationship holds under conditions in which (i) almost all excitation photons are absorbed within the blood sample and (ii) the detection efficiency for fluorescence photons is virtually independent of the red blood cell concentration. Due to intense blood absorption in the blue spectral range, almost all excitation photons are absorbed by the blood sample within a sufficiently thin layer below the sample surface to allow the fluorescence photons emitted through the surface to be detected with equal efficiency. Even large variations of the red blood cell concentration vary the penetration depth of the excitation photons only slightly. The conventional hematofluorometer used for detection is insensitive to such small variations.

Under these conditions, the detected fluorescence intensity is approximately proportional to the molar *ratio* of the concentration of zinc protoporphyrin to the concentration of hemoglobin. Since virtually all excitation photons are absorbed within the blood sample, the probability for emission of a zinc protoporphyrin fluorescence photon upon absorption of an excitation photon is proportional to the contribution of zinc protoporphyrin absorption to the total absorption,

$$F_{\mathrm{ZnPP}} \propto \frac{\mu_{\mathrm{a,ZnPP}}}{\mu_{\mathrm{a,Hb}} + \mu_{\mathrm{a,ZnPP}}} \quad (1)$$

where F_{ZnPP} is the detected fluorescence intensity, and $\mu_{\mathrm{a,ZnPP}}$ and $\mu_{\mathrm{a,Hb}}$ are the absorption coefficients of zinc protoporphyrin and hemoglobin, respectively. Within the red blood cell, hemoglobin, with a concentration that is some five orders of magnitude greater

than that of zinc protoporphyrin, accounts for almost all the absorbance, leaving the absorbance of zinc protoporphyrin negligible. Accordingly, equation 1 can be simplified to

$$F_{ZnPP} \propto \frac{\mu_{a,\,ZnPP}}{\mu_{a,\,Hb} + \mu_{a,\,ZnPP}} \approx \frac{\mu_{a,\,ZnPP}}{\mu_{a,\,Hb}} \quad (2)$$

In turn, the absorption coefficients of zinc protoporphyrin and hemoglobin are proportional to their respective molar concentrations. Consequently, the zinc protoporphyrin fluorescence is proportional to the molar ratio of the concentration of zinc protoporphyrin to the concentration of hemoglobin.

$$F_{ZnPP} \propto \frac{c_{ZnPP}}{c_{Hb}} \quad (3)$$

The proportionality of equation 3 also holds for fiber-based fluorescence measurements on blood. Beneath the fiber optic probe, almost all excitation photons are absorbed within a sufficiently thin layer of blood to allow the fluorescence photons emitted to be detected with equal efficiency. As shown in Fig. 3*a*, the detected zinc protoporphyrin fluorescence is nearly independent of the red blood cell concentration for a concentration between 2% and 8%.

For the fiber-based fluorescence measurements on tissue, other tissue parameters also influence the validity of equation 3. In particular, sites with a blood volume fraction that is too low, an average blood vessel diameter that is too large or an excessively thick epithelial layer must be identified and excluded. By excluding sites with a blood absorption index $<0.7 \times 10^{-2}$, tissue sites were excluded where the measured zinc protoporphyrin fluorescence deviated substantially from the expected value (see main Fig. 3 for the blood absorption index and Supplementary Fig. 6 for Monte Carlo simulations). Consequently, measurements were started in the clinical study reported here only when the blood absorption index exceeded the lower threshold of 0.7×10^{-2} for the blood absorption index. In a preparatory study, a number of tissue sites could be found with a blood absorption index $\geq 0.7 \times 10^{-2}$ in all subjects.

Influence of blood properties on the non-invasive measurements. The excitation wavelengths 425 nm and 407 nm were chosen such that blood absorption is virtually identical (Fig. 1*c*). Changes in blood volume fraction and influences by packaging effects, including variable vessel size, modify the absorption at both excitation wavelengths equally. None of these effects influences the molar *ratio* of the concentration of zinc protoporphyrin to the concentration of hemoglobin so that the emitted zinc protoporphyrin fluorescence remains constant (see equation 3).

For the blood absorption spectrum used for the selection of the two excitation wavelengths, an average hemoglobin oxygenation of 85% was assumed (Supplementary Fig. 3). The average hemoglobin oxygenation found in tissue may vary for different locations for the same patient or between patients. Consequently, other average hemoglobin oxygenation states (75%-100%) were studied as well (Supplementary Fig. 3). Due to the isosbestic points in the absorption spectra of oxygenated and deoxygenated hemoglobin at 390 nm and 422 nm,[1] the absorption coefficient varies for 425 nm and 407 nm only by 1% each within the considered range of average hemoglobin oxygenation. Consequently, for 425 nm excitation, the zinc protoporphyrin fluorescence signal would be influenced by less than 1% as well, since it is inversely proportional to the hemoglobin absorption (see equation 2). Larger variations in the fluorescence background are expected between the two excitation wavelengths for different hemoglobin oxygenation states. The absorption coefficients for the excitation wavelengths differ by up to 2%, leading to a different penetration depth and therefore exciting different background fluorophores. Nonetheless, for calculation of the difference spectrum, the 407 nm background spectrum is scaled to the 425 nm spectrum, thereby eliminating most of the variations. The remaining variations can be assumed to yield a slightly varying but still smooth remaining background in the difference spectrum, which is then eliminated by the spectral fitting algorithm.

Hemoglobin variants might influence the non-invasive erythrocyte zinc protoporphyrin measurements if their absorption properties were to differ at the blue excitation wavelengths, at the absorption maximum around 576 nm used for determining the blood absorption index,

or both. For fetal hemoglobin (Hb F) and sickle hemoglobin (Hb S), the absorption spectra of both oxygenated and deoxygenated forms are virtually identical to hemoglobin A (Hb A) absorption spectra in the visible spectral range.[4-6] Consequently, these common hemoglobin variants are not expected to influence the erythrocyte zinc protoporphyrin measurements.

Choice of fiber diameter. The choice of the fiber diameter for fluorescence excitation and detection was influenced by two considerations. First, the amount of fluorescence photons emitted by zinc protoporphyrin preferably should be independent of the unknown tissue parameters that vary within and among subjects. Second, as many zinc protoporphyrin fluorescence photons as possible should be collected for reliable measurements. As evident from Supplementary Fig. 5*a*, *b* and *d*, the influence of a varying blood volume fraction, average blood vessel diameter and epithelial thickness becomes smaller for thicker optical fibers. In Supplementary Fig. 5*c*, the smallest influence of a varying μ_s' is found for the 1000 µm fiber. The influence of a varying reduced scattering coefficient μ_s', is less pronounced than for a varying blood volume fraction and epithelial thickness. In Supplementary Fig. 5*e*, the amount of detected zinc protoporphyrin photons is compared for different fiber diameters, with the output power limited by the area of application to comply with the light exposure limits defined by the international standard IEC 60825-1:2014. To maximize the amount of detected fluorescence photons, a larger fiber diameter is preferable. Taking these considerations into account, a 1000 µm diameter optical fiber was chosen. For this diameter, the influence of the different tissue parameters is small and the amount of detected zinc protoporphyrin fluorescence photons is still reasonably high. Compared to the 1500 µm fiber, the 1000 µm fiber is much more flexible and readily used during the measurement procedure.

Supplementary Note 2. Outliers in the evaluation in women after childbirth

In the Bland-Altman plot in Fig. 5*b*, comparing non-invasive measurements *in vivo* with HPLC results *in vitro*, two possible outliers can be identified (subjects #44 and 45 in Supplementary Table 1). The remaining 54 data points in the Bland-Altman plot are normally distributed but if all 56 points are included their distribution is no longer normal (Shapiro-Wilk test, software: R version 3.2.0, function: *shapiro.test*). The two possible outliers are also present if the conventional hematofluorometer measurements on whole blood are compared with the HPLC results (see Supplementary Table 1). By contrast, neither possible outlier is present if the non-invasive measurements *in vivo* are compared with the conventional hematofluorometer measurements on whole blood *in vitro* (see Supplementary Table 1), suggesting a systematic difference between the HPLC and optical measurements for subjects #44 and 45. For subject #45 (original value 138.1 µmol/mol heme), a repeat HPLC measurement found a value of 63.2 µmol/mol heme that would no longer be considered an outlier. For subject #44 (original value 20.5 µmol/mol heme), the repeat HPLC measurement was similar (24.1 µmol/mol heme; Supplementary Fig. 7). No unusual medication was found for this subject. The explanation for the divergence in the HPLC and optical measurements for subject #44 remains to be determined.

Supplementary Note 3. Screening for iron deficiency with the zinc protoporphyrin/ heme ratio

The sensitivity and specificity of all methods of screening for iron deficiency by measuring the zinc protoporphyrin/heme ratio will depend upon the prevalence in the population examined of other conditions that elevate the ratio in iron-replete individuals. These include a variety of genetic and acquired disorders, including certain porphyrias[7], compound heterozygotes for mutations in the gene KLF1 (Kruppel-Like Factor 1 (Erythroid))[8], some forms of myelodysplasia[9] and a variety of sideroblastic and inherited microcytic anemias.[10] Because these disorders are uncommon or rare in all populations, such conditions would

have virtually no effect on the use of the zinc protoporphyrin/heme ratio for screening. In contrast, because lead and other heavy metals[11,12] increase erythrocyte zinc protoporphyrin independently of iron status, the ratio is of little use in populations exposed to these toxins. In populations with an appreciable prevalence of hemoglobinopathies, the effects on the sensitivity and specificity will depend on the specific hemoglobin abnormalities and their frequency. Erythrocyte zinc protoporphyrin levels are normal in sickle-cell trait but elevated in sickle-cell anemia in the subpopulation that has low fetal hemoglobin levels (<9%).[13] Nonetheless, the erythrocyte zinc protoporphyrin/heme ratio has been used to screen patients with sickle cell anemia for iron deficiency.[14] Some studies found no elevations in erythrocyte protoporphyrins with α-thalassemia trait[15] or β-thalassemia trait[16] but others have reported modest elevations in subpopulations of iron-replete individuals with α-thalassemia, β-thalassemia and with hemoglobin E that overlap with levels found in mild iron deficiency.[17-20] Depending upon the prevalence of these hemoglobinopathies in the population examined, the sensitivity and specificity of the erythrocyte zinc protoporphyrin/heme ratio for iron deficiency will be altered. Although recurrent acute infections[21] and chronic inflammation[22] can raise the erythrocyte zinc protoporphyrin/heme ratio in iron-replete individuals, the World Health Organization nonetheless recommends red blood cell zinc protoporphyrin as the preferred indicator of iron deficiency in children in regions endemic for malaria and other infections.[23,24] The follow up of individuals identified by screening will depend upon the screening program and population examined. In clinical care, referral to a physician for further evaluation may be necessary.[25] In other circumstances, such as during pregnancy,[26] screening regular blood donors for pre-anemic iron deficiency[27] or screening to target iron supplementation in populations where iron deficiency is overwhelmingly nutritional,[28] a therapeutic trial of iron may be indicated. Erythrocyte protoporphyrin is also used to screen for iron deficiency in epidemiological surveys[29-31] and in the evaluation of programs of iron fortification and supplementation.[32-38]

SUPPLEMENTARY REFERENCES

1. Prahl, S. Optical Properties Spectra. <http://omlc.org/spectra/hemoglobin/> (2003).
2. Finlay, J. C. & Foster, T. H. Effect of pigment packaging on diffuse reflectance spectroscopy of samples containing red blood cells. *Opt Lett* **29**, 965-967 (2004).
3. Blumberg, W. E., Eisinger, J., Lamola, A. A. & Zuckerman, D. M. The hematofluorometer. *Clin Chem* **23**, 270-274 (1977).
4. De Llano, J. J. M. & Manning, J. M. Properties of a recombinant human hemoglobin double mutant: Sickle hemoglobin with Leu 88 at the primary aggregation site substituted by Ala. *Protein Sci* **3**, 1206-1212 (1994).
5. White, J. C. & Beaven, G. H. A review of the varieties of human haemoglobin in health and disease. *J Clinical Path* **7**, 175-200 (1954).
6. Zijlstra, W. G., Buursma, A. & Meeuwsen-Van der Roest, W. P. Absorption spectra of human fetal and adult oxyhemoglobin, de-oxyhemoglobin, carboxyhemoglobin, and methemoglobin. *Clin Chem* **37**, 1633-1638 (1991).
7. Besur, S., Hou, W., Schmeltzer, P. & Bonkovsky, H. L. Clinically important features of porphyrin and heme metabolism and the porphyrias. *Metabolites* **4**, 977-1006 (2014).
8. Satta, S. *et al.* Compound heterozygosity for KLF1 mutations associated with remarkable increase of fetal hemoglobin and red cell protoporphyrin. *Haematologica* **96**, 767-770 (2011).
9. Hastka, J., Lasserre, J. J., Schwarzbeck, A., Strauch, M. & Hehlmann, R. Zinc protoporphyrin: a screening parameter of iron metabolism. *Onkologie* **14**, 62-63 (1991).
10. Iolascon, A., De Falco, L. & Beaumont, C. Molecular basis of inherited microcytic anemia due to defects in iron acquisition or heme synthesis. *Haematologica* **94**, 395-408 (2009).
11. Labbe, R. F., Vreman, H. J. & Stevenson, D. K. Zinc protoporphyrin: A metabolite with a mission. *Clin Chem* **45**, 2060-2072 (1999).
12. Martin, C. J., Werntz, C. L. & Ducatman, A. M. The interpretation of zinc protoporphyrin changes in lead intoxication: a case report and review of the literature. *Occup Med*

(Lond) **54**, 587-591 (2004).

13. Hirsch, R. E., Pulakhandam, U. R., Billett, H. H. & Nagel, R. L. Blood zinc protoporphyrin is elevated only in sickle cell patients with low fetal hemoglobin. *Am J Hematol* **36**, 147-149 (1991).
14. Mohanty, D. *et al.* Iron deficiency anaemia in sickle cell disorders in India. *Indian J Med Res* **127**, 366-369 (2008).
15. Koenig, H. M., Lightsey, A. L. & Schanberger, J. E. The micromeasurement of free erythrocyte protoporphyrin as a means of differentiating alpha thalassemia trait from iron deficiency anemia. *J Pediatr* **86**, 539-541 (1975).
16. Stockman, J. A., Weiner, L. S., Simon, G. E., Stuart, M. J. & Oski, F. A. The measurement of free erythrocyte porphyrin (FEP) as a simple means of distinguishing iron deficiency from beta-thalassemia trait in subjects with microcytosis. *J Lab Clin Med* **85**, 113-119 (1975).
17. Tillyer, M. L. & Tillyer, C. R. Zinc protoporphyrin assays in patients with alpha and beta thalassaemia trait. *J Clin Pathol* **47**, 205-208 (1994).
18. Hinchliffe, R. F., Lilleyman, J. S., Steel, G. J. & Bellamy, G. J. Usefulness of red cell zinc protoporphyrin concentration in the investigation of microcytosis in children. *Pediatr Hematol Oncol* **12**, 455-462 (1995).
19. Graham, E. A., Felgenhauer, J., Detter, J. C. & Labbe, R. F. Elevated zinc protoporphyrin associated with thalassemia trait and hemoglobin E. *J Pediatr* **129**, 105-110 (1996).
20. Hinchliffe, R. F., Vora, A. J. & Lennard, L. An assessment of methods used in the investigation of iron status: findings in a population of young British South Asian children. *J Clin Pathol* (2015).
21. Crowell, R., Ferris, A. M., Wood, R. J., Joyce, P. & Slivka, H. Comparative effectiveness of zinc protoporphyrin and hemoglobin concentrations in identifying iron deficiency in a group of low-income, preschool-aged children: practical implications of recent illness. *Pediatrics* **118**, 224-232 (2006).

22. Hastka, J., Lasserre, J. J., Schwarzbeck, A., Strauch, M. & Hehlmann, R. Zinc protoporphyrin in anemia of chronic disorders. *Blood* **81**, 1200-1204 (1993).

23. WHO. Conclusions and recommendations of the WHO Consultation on prevention and control of iron deficiency in infants and young children in malaria-endemic areas. *Food Nutr Bull* **28**, S621-7 (2007).

24. WHO. Report of the World Health Organization Technical Consultation on Prevention and Control of Iron Deficiency in Infants and Young Children in Malaria-Endemic Areas, Lyon, France, 12-14 June 2006. *Food Nutr Bull* **28**, S489-631 (2007).

25. Brittenham, G. M. in *Hematology: Basic Principles and Practice* (eds Hoffman, R. *et al.*) 437-449 (Elsevier, New York, 2013).

26. Schifman, R. B., Thomasson, J. E. & Evers, J. M. Red blood cell zinc protoporphyrin testing for iron-deficiency anemia in pregnancy. *Am J Obstet Gynecol* **157**, 304-307 (1987).

27. Jensen, B. M., Sando, S. H., Grandjean, P., Wiggers, P. & Dalhoj, J. Screening with zinc protoporphyrin for iron deficiency in non-anemic female blood donors. *Clin Chem* **36**, 846-848 (1990).

28. Zimmermann, M. B. & Hurrell, R. F. Nutritional iron deficiency. *Lancet* **370**, 511-520 (2007).

29. Cogswell, M. E. *et al.* Assessment of iron deficiency in US preschool children and nonpregnant females of childbearing age: National Health and Nutrition Examination Survey 2003-2006. *Am J Clin Nutr* **89**, 1334-1342 (2009).

30. Mei, Z., Parvanta, I., Cogswell, M. E., Gunter, E. W. & Grummer-Strawn, L. M. Erythrocyte protoporphyrin or hemoglobin: which is a better screening test for iron deficiency in children and women? *Am J Clin Nutr* **77**, 1229-1233 (2003).

31. Mei, Z. *et al.* Assessment of iron status in US pregnant women from the National Health and Nutrition Examination Survey (NHANES), 1999-2006. *Am J Clin Nutr* **93**, 1312-1320 (2011).

32. Andersson, M. *et al.* Dual fortification of salt with iodine and iron: a randomized,

double-blind, controlled trial of micronized ferric pyrophosphate and encapsulated ferrous fumarate in southern India. *Am J Clin Nutr* **88**, 1378-1387 (2008).

33. Baumgartner, J. *et al.* Overweight impairs efficacy of iron supplementation in iron-deficient South African children: a randomized controlled intervention. *Int J Obes (Lond)* **37**, 24-30 (2013).
34. Blanco-Rojo, R. *et al.* Efficacy of a microencapsulated iron pyrophosphate-fortified fruit juice: a randomised, double-blind, placebo-controlled study in Spanish iron-deficient women. *Br J Nutr* **105**, 1652-1659 (2011).
35. Muthayya, S. *et al.* Iron fortification of whole wheat flour reduces iron deficiency and iron deficiency anemia and increases body iron stores in Indian school-aged children. *J Nutr* **142**, 1997-2003 (2012).
36. Olney, D. K. *et al.* Combined iron and folic acid supplementation with or without zinc reduces time to walking unassisted among Zanzibari infants 5- to 11-mo old. *J Nutr* **136**, 2427-2434 (2006).
37. Stoltzfus, R. J. *et al.* Low dose daily iron supplementation improves iron status and appetite but not anemia, whereas quarterly anthelminthic treatment improves growth, appetite and anemia in Zanzibari preschool children. *J Nutr* **134**, 348-356 (2004).
38. Zimmermann, M. B. *et al.* Addition of microencapsulated iron to iodized salt improves the efficacy of iodine in goitrous, iron-deficient children: a randomized, double-blind, controlled trial. *Eur J Endocrinol* **147**, 747-753 (2002).
